# Exploratory study of dual‐task digital device in children and adolescents with attention‐deficit/hyperactivity disorder

**DOI:** 10.1002/pcn5.70089

**Published:** 2025-04-02

**Authors:** Katsunaka Mikami, Tasuku Miyajima, Ryo Nishino, Naohiro Kawazoe, Toshimitsu Ochiai, Takashi Okada, Hiroki Fukuju

**Affiliations:** ^1^ Department of Psychiatry Tokai University School of Medicine Kanagawa Japan; ^2^ Tokyo Kasei University Tokyo Japan; ^3^ Institute for Advancement of Clinical and Translational Science (iACT) Kyoto University Hospital Kyoto Japan; ^4^ Drug Development and Regulatory Science Division, Shionogi & Co., Ltd. Osaka Japan; ^5^ Biostatistics Center, Shionogi & Co., Ltd. Osaka Japan; ^6^ Department of Psychiatry Nara Medical University Nara Japan

**Keywords:** attention deficit hyperactivity disorder, children, cognitive function, digital device, Japan

## Abstract

**Aim:**

The study aimed to assess the efficacy and safety of SDT‐001, a digital therapeutic, in Japanese children and adolescents with attention‐deficit/hyperactivity disorder (ADHD).

**Methods:**

This phase 2, multicenter, randomized, double‐blind, sham‐controlled study (jRCT1080225158) was conducted for a duration of up to 14 weeks. After screening, eligible participants were randomized to receive SDT‐001 or single‐task intervention for 25 min/day for 6 weeks and followed for 4 weeks after the intervention. A post hoc analysis was also performed to compare the effects of SDT‐001 or a single‐task to a nonrandomized, open‐labeled, observational group (as the reference follow‐up group; without single‐ or dual‐task training; and continuing psychosocial treatment, including environmental adjustment).

**Results:**

Overall, 262 participants were enrolled in the study between July 2020 and July 2021. Of these, 261 participants were included in the analysis (SDT‐001, *n* = 108; single‐task, *n* = 107; observation, *n* = 46). ADHD Rating Scale‐IV (ADHD‐RS‐IV) scores (physician's assessment) decreased gradually, with greater reduction in SDT‐001 versus the single‐task group at week 6 (ADHD‐RS‐IV‐Total score, least‐squares mean change from baseline [95% confidence interval] −7.5 [9.0, −6.1] vs. −6.5 [−7.9, −5.0], P = 0.2112). Reductions in inattention and hyperactivity/impulsivity scores were maintained during 4‐week follow‐up after treatment completion. At week 6, the scores of both groups improved compared to the nonrandomized observation group in post hoc analysis.

**Conclusion:**

These findings suggest SDT‐001 as a promising treatment option, addressing the challenges of psychosocial treatment and pharmacotherapy in Japanese children and adolescents with ADHD.

## Graphical abstract

This study aimed to assess the efficacy and safety of SDT‐001, a digital therapeutic, in Japanese children and adolescents with attention‐deficit/hyperactivity disorder (ADHD). The SDT‐001 group tended to show a greater change from baseline in the ADHD‐RS‐IV scores than the single‐task group, but this difference was not statistically significant compared to the single‐task group. Post hoc analysis comparing with the observation group suggested SDT‐001 as a promising treatment option for treating ADHD symptoms.

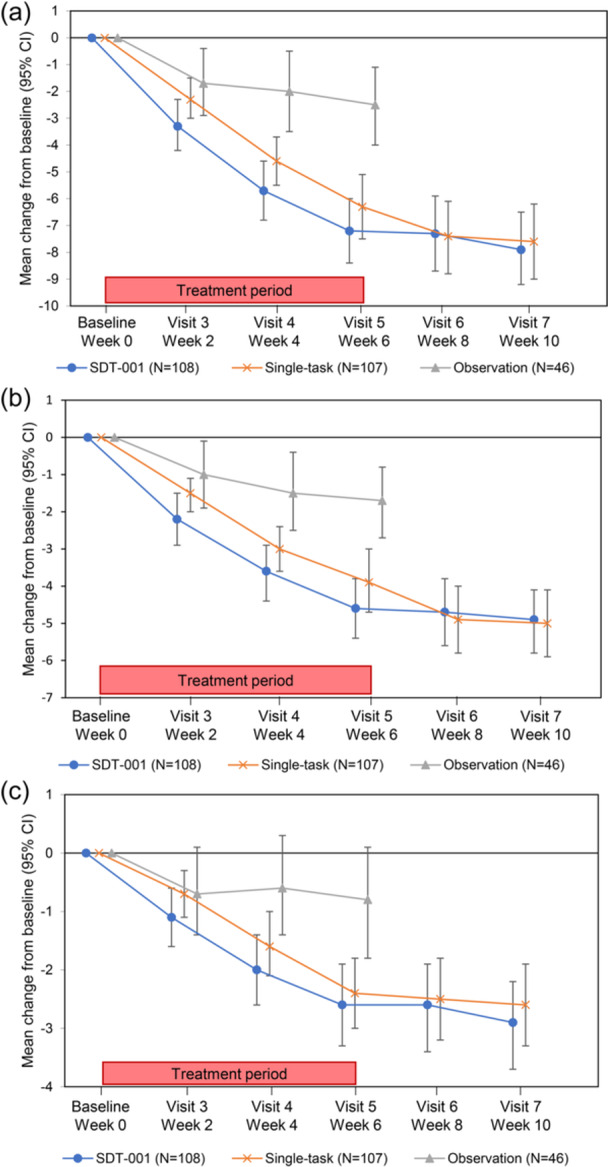

## INTRODUCTION

Attention‐deficit/hyperactivity disorder (ADHD) is a common neurodevelopmental disorder characterized by hyperactivity‐impulsivity and/or inattention, typically diagnosed in children and adolescents, and often persisting into adulthood.^1^ Globally, the estimated prevalence of ADHD was 7.6% in children aged 3–12 years and 5.6% in those aged 12–18 years.^1^ In Japan, a total of 838,265 individuals were newly diagnosed with ADHD between fiscal years (FY) 2010 and 2019, of which 121,278 (14.47%) were diagnosed at age 0–6 years, 381,753 (45.54%) at age 7–19 years, and 335,234 (39.99%) after age 19.^2^

In ADHD, motor hyperactivity tends to decrease with age, and symptoms of inattention and impulsivity often persist into adulthood.^3^, ^4^ ADHD is associated with maladjustment and mental health issues that often require treatment.^3^, ^5^ Japanese guidelines recommend environment adjustment and psychosocial interventions, with pharmacological therapy added if necessary.^6^, ^7^ However, the evidence showed variation between clinicians/therapists in the effectiveness of psychosocial therapy.^8^, ^9^ Moreover, variability in the effectiveness of psychosocial therapy due to suboptimal engagement with care and discontinuation of medications owing to side effects and addiction risk are reported as barriers for effective ADHD treatment in children.^10^ These findings emphasize the need for a new treatment option for the management of ADHD.

Cognitive deficits may result from impaired neural processes for cognitive control. Gazzaley et al. demonstrated that personalized dual‐task digital intervention can improve executive function.^11^ Digital therapeutics (DTx) deliver medical interventions via health software, with demonstrable positive impact (https://dtxalliance.org/). In Japan, DTx are recognized as medical devices. Interventions with DTx offer versatile approaches, including standalone or combined therapies, or hardware‐assisted therapies like neurofeedback training. Additionally, DTx has been shown to improve attention, working memory (WM), and inhibition in children with ADHD,^12^ and multitasking in healthy older adults.^13^ The evidence also showed the positive effect of nonpharmacological interventions such as neurofeedback and cognitive‐behavioral therapy on self‐reported and parent–teacher‐reported ADHD cognitive symptomatology^14^ and suggested that DTx can be an effective and promising therapy in improving various aspects of ADHD symptoms in children and adolescents.^15^

The United States Food and Drug Administration has approved EndeavorRx® (AKL‐T01), the first DTx for treatment of ADHD in children aged 8–17 years. Clinical studies of AKL‐T01 in children with ADHD showed improvements in attention and impulsivity, with minimal adverse events (AEs).^16^, ^17^ In the STARS‐ADHD study, AKL‐T01 significantly improved Test of Variables of Attention® (TOVA®) Attention Performance Index scores (P < 0.0001) compared to a control intervention that was designed to match AKL‐T01 on expectancy, engagement, and time spent, using a challenging and engaging digital word game, targeting cognitive domains. However, the study included children not taking ADHD medication and a single 4‐week treatment regimen as intervention.^17^ In the STARS‐Adjunct study, combining AKL‐T01 with pharmacotherapy significantly improved Impairment Rating Scale (IRS) scores (P < 0.001) over two 4‐week treatment periods and suggested superior academic and conduct outcomes.^16^

The SDT‐001 is an application that translates the language and expressions used in EndeavorRx® into Japanese. This phase 2, randomized, double‐blind, sham‐controlled study was conducted to assess the efficacy and safety of SDT‐001, a dual‐task training device, in Japanese children and adolescents with ADHD. In addition to the above purposes, this study aimed to validate single‐task training as the “sham candidate” to achieve indistinguishability and functional dissimilarity to ensure blindness.^13^, ^18^ A post hoc analysis was also performed to compare the effects of SDT‐001 and the single task to a nonrandomized, open‐labeled, observational group (as the reference follow‐up group; without single‐ or dual‐task training; and continuing psychosocial treatment, including environmental adjustment, that was in place during the study consent).

## METHODS

### Study design

**Figure 1:**
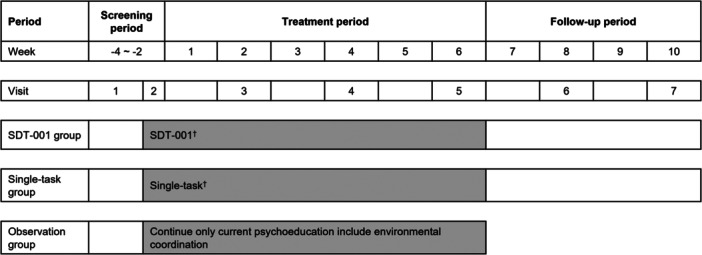
Study design. Schematic depicting the randomized, double‐blind, sham‐controlled study. The study consisted of three periods, that is, the screening period for 2–4 weeks, the treatment period for 6 weeks, and the follow‐up period for 4 weeks. ^†^Patient continues the treated psychoeducation including environmental coordination at the time of signing the informed consent form. SDT‐001, investigational digital therapeutic.

This was a phase 2, multicenter, randomized, double‐blind, sham‐controlled study to assess the safety and efficacy of SDT‐001, a programmed medical device, in children and adolescents with ADHD. The study was conducted from July 2020 to July 2021 at 64 medical institutions in Japan (trial registration no.: jRCT1080225158). The study consisted of up to 14 weeks divided into three periods: −4 to −2 weeks for screening, 6 weeks of treatment, and 4 weeks of posttreatment follow‐up (Figure 1). A nonrandomized, open‐label reference group of participants who continued psychosocial treatment (including environmental adjustment) was also observed for 6 weeks, but no follow‐up was conducted for the observation group. After screening, the eligible participants were randomized in a double‐blinded manner to SDT‐001 or single‐task (sham candidate without task difficulty adjustment) groups in a ratio of 1:1 by the stochastic minimization method, with the presence/absence of a history of pharmacotherapy indicated for ADHD and age (≤12 or ≥13 years at informed consent) as allocation factors. Participants were instructed to use the study device for approximately 25 min daily for 7 days/week for 6 weeks (Table 1). Participants were assessed at baseline (prior to interventions) and once every 2 weeks until follow‐up.

**Table 1:** Device specifications.

Study group	SDT‐001 group	Single‐task group
Name of study device	SDT‐001	Sham^a^
Treatment frequency and duration	Once daily for 6 weeks (25 min)	Once daily for 6 weeks (25 min)
Use	Investigational device	Control device
Specifications	Dual tasks The task difficulty level was automatically optimized for each patient's level	Single task A certain task difficulty level was set, and the level was not adjusted according to each patient's level

*Note*: SDT‐001 and single‐task devices used during this study were manufactured by Akili Interactive Labs, Inc. (MA, USA). The devices were installed in iPad mini® in advance and then provided to the medical institutions.

Abbreviation: SDT‐001, investigational digital therapeutic.

^a^ Software program (application) the same as SDT‐001 without the core mechanisms of SDT‐001.

### Randomization and blinding

Participants were centrally randomized by the Interactive Web Response System. Except for the observation group, this study was conducted as a double‐blind study with a single‐task (sham candidate) device that is indistinguishable in appearance, labeling, and packaging. The SDT‐001 and single‐task interventions were pre‐installed in iPad mini tablets (Apple, Japan) labelled as “study devices” at the device management vendor, based on the study device randomization performed by the assignment manager. Participants accessed their randomized intervention with a unique username and password. The blinding remained intact for all individuals except for the assignment manager and the kitting manager until after data lock. The blinding was ensured by taking the following measures throughout the study: siblings of eligible participants were not included in the study to prevent comparison of devices between participants within families at the time of recruitment; detailed explanation of the study device (e.g., core mechanism) was not provided in the informed consent/assent form and the written information; and description that might identify the SDT‐001 or single‐task groups was not provided at the time of the informed consent explanation. An independent contact center was established from the study site and the sponsor for inquiries about deficiencies in the study device and ensured that the investigator (subinvestigator), study coordinator, or the sponsor did not receive direct inquiries about the contents of the study device from participants and/or their legally authorized representatives. The study device was set “not accessible” by the investigator (subinvestigator), study coordinator, or sponsor. At study conclusion, all the devices were collected, and login control was used to prevent the investigators, including the site staff and the sponsor, from accessing the applications in the devices. Additionally, the study included a questionnaire to evaluate blinding for each group.

### Study participants

Study eligibility criteria are summarized in Table S1. The study included male or female outpatients who were pupils and students aged 6–17 years, with confirmed diagnosis of ADHD based on Diagnostic and Statistical Manual of Mental Disorders 5th edition and an ADHD Rating Scale‐IV (ADHD‐RS‐IV) inattentive subscale score (physician's assessment) of ≥15 points (at both screening and baseline); who received psychosocial treatment (including environmental adjustment) for ADHD for an adequate period that was considered not to have had sufficient effect; and those that had not received pharmacotherapy for ADHD within 7 days prior to the enrollment. The observation group enrolled participants who had never received pharmacotherapy for ADHD.

Children and adolescents with change (from baseline to screening) in the ADHD‐RS‐IV inattentive subscale score >30%; suspected gaming disorder; suicidal tendency or suspected substance‐related disorder within 180 days prior to the screening; psychiatric diseases such as schizophrenia spectrum, depression, bipolar disorder, personality disorder, or intellectual disability; convulsion or severe tic disorder (including Tourette's disorder); or who were unable to undergo assessments with the study device and study‐specific activities for physical reasons were excluded. Children and adolescents who used any prohibited concomitant drugs/therapies (including prescription drugs, over‐the‐counter drugs, and health foods) between screening to baseline or received any additional psychosocial treatments (including environmental adjustment) or change in the conditions of treatment or with previous participation by themselves/their siblings were also excluded.

### Study device

The study devices, SDT‐001 and single‐task (sham candidate), were manufactured by Akili Interactive Labs, Inc. (MA, USA). An overview of the study devices is given in Table 1. The SDT‐001 is an investigational DTx that uses a proprietary algorithm designed to improve not only attention but also ADHD by performing dual‐tasks at personalized difficulty levels. Details of SDT‐001 (AKL‐T01) were previously published by Kollins et al.^17^ The single‐task (sham candidate) control was designed to match and maintain blinding to SDT‐001 in this study. The sham device was developed with a single task but the activity elements and appearance were similar to those of SDT‐001 to ensure blindness. Performance on each task was assessed on a single task only, and a target level of training was set according to the individual's performance level. The level of difficulty was fixed for the training, and the user could advance to the next stage uniformly after a certain number of training sessions.

### Study endpoints and assessments

The efficacy endpoints were change from baseline (visit 2) to week 6 (visit 5) in scores on ADHD‐RS‐IV (Total [ADHD‐RS‐T], Inattentive [ADHD‐RS‐I], Hyperactive/Impulsive [ADHD‐RS‐H] subscale scores; physician's assessment); TOVA® (using visual stimulation test) attention comparison scores (ACS); IRS; Conners 3™ for parents; clinical global impression‐improvement (CGI‐I) (as proportion of participants assessed as “very much improved” or “much improved”); and physician's global assessment (PGA) (rate of participants assessed as “very much improved” or “much improved”).

The ADHD‐RS‐IV is an 18‐item assessment for core symptoms of ADHD on a four‐point rating scale (0–3, the odd criteria for inattention, and the even criteria for hyperactivity/impulsivity).^17^, ^19^ In this study, the Japanese version of ADHD‐RS‐IV was used.^20^

The TOVA®, a validated, computerized, objective continuous performance test, with ACS represents comparison of participant's performance for ADHD and non‐ADHD. Of the visual and auditory stimulation tests in TOVA®, this study used the visual stimulation tests.^21^

The IRS is an eight‐item assessment for functional impairment or for assessing the impact of individualized areas of impairment on a child's functioning across a range of domains via a visual analog scale.^22^

The Conners 3™ is a 110‐item assessment scale used by parents for assessment of ADHD‐related symptoms.^23^ The CGI‐I is a clinician‐administered assessment of improvements in global impressions of ADHD severity on a seven‐point scale.^24^ The PGA is an indicator for overall assessment of ADHD‐related symptoms.

The safety endpoints, AEs, adverse device effects, Columbia‐Suicide Severity Rating Scale (C‐SSRS), and questionnaire about gaming addiction were reported during each assessment. Among the AEs reported in the treatment period, those related to the study device were regarded as adverse device effects. The C‐SSRS was used to evaluate the presence or absence of suicidal ideation and suicidal behavior during each assessment.

### Sample size

In this study, the sample size was not determined based on statistical power. The sample size of 90 was chosen for both the SDT‐001 and single‐task groups to provide estimated precision, resulting in half of the width of a 95% confidence interval (CI) for the between‐group difference with a range from 1.5 to 2.5. This estimation assumes a standard deviation (SD) of 5–8 for changes in ADHD‐RS‐IV scores (total and subscale) from baseline. In addition, the sample size of 30 participants in the observation group was considered feasible.

### Statistical analysis

Descriptive statistics were used to summarize demographics and baseline characteristics for participants in each study group. Categorical variables were reported as numbers (*n*) and percentages (%), and continuous variables as mean and SD. Statistical tests were performed with a two‐sided significance level of 0.05 and all CIs were estimated as two‐sided unless otherwise stated. All statistical analyses were performed using SAS (version 9.4) (SAS Institute, Cary, NC, USA).

Unless otherwise stated, efficacy analyses were performed on the full analysis set, which included all participants who were randomly assigned to the SDT‐001 or single‐task group and used the study device, and those who were enrolled in the observation group in the treatment period.

For ADHD‐RS‐IV total and subscale scores (physician's assessment), TOVA® ACS, and IRS, the between‐group difference and its 95% CI were calculated using a mixed‐effects model repeated measures method. A model with unstructured covariance structure on error variance was applied to data obtained at each time point of assessment at visits three to five, using the change from baseline as a response variable; the group, assessment time point, and interaction between the group and assessment time point as fixed effects; and baseline values, age group, and presence/absence of history of pharmacotherapy indicated for ADHD as covariates.

For Conners 3™, the analysis of covariance for change from baseline to week 6 was performed, and between‐group differences and 95% CIs were calculated with the baseline values, age group, and presence/absence of history of pharmacotherapy indicated for ADHD as covariates.

For CGI‐I and PGA, the number and proportion of responders were summarized for each group, and the 95% CI of proportion estimated with the Clopper–Pearson method. The difference in the proportion of responders between treatment groups and its 95% CI was estimated by the Cochran–Mantel–Haenszel method stratified by age group and the presence/absence of history of pharmacotherapy indicated for ADHD.

Post hoc analysis was performed to investigate the efficacy in the SDT‐001 or single‐task groups compared to the non‐randomized observation group. The inverse probability weighting analysis using propensity score was applied with the intention of correcting the bias in baseline subject characteristics between groups. The propensity score was calculated by adjustment of baseline subject characteristics: age, sex, ADHD type, and baseline value of the ADHD‐RS‐IV (physician's assessment) inattention score. This analysis was performed for the set of participants who had no treatment history with drugs indicated for ADHD in the SDT‐001, single‐task, and observation groups.

Safety analyses were performed for all participants who were randomly assigned to the SDT‐001 or the single‐task group, used the study device, and were analyzed based on the actual device used, and those who were enrolled in the observation group in the treatment period. The AEs that occurred after enrollment in the treatment period were used for safety analyses, and the incidences were summarized by group.

### Study ethics

This study was conducted in compliance with the consensus framework for ethical principles, applicable laws and regulations. The study was approved by the Institutional Review Boards/Independent Ethics Committees (Table S2).

## RESULTS

### Patient disposition

**Figure 2:**
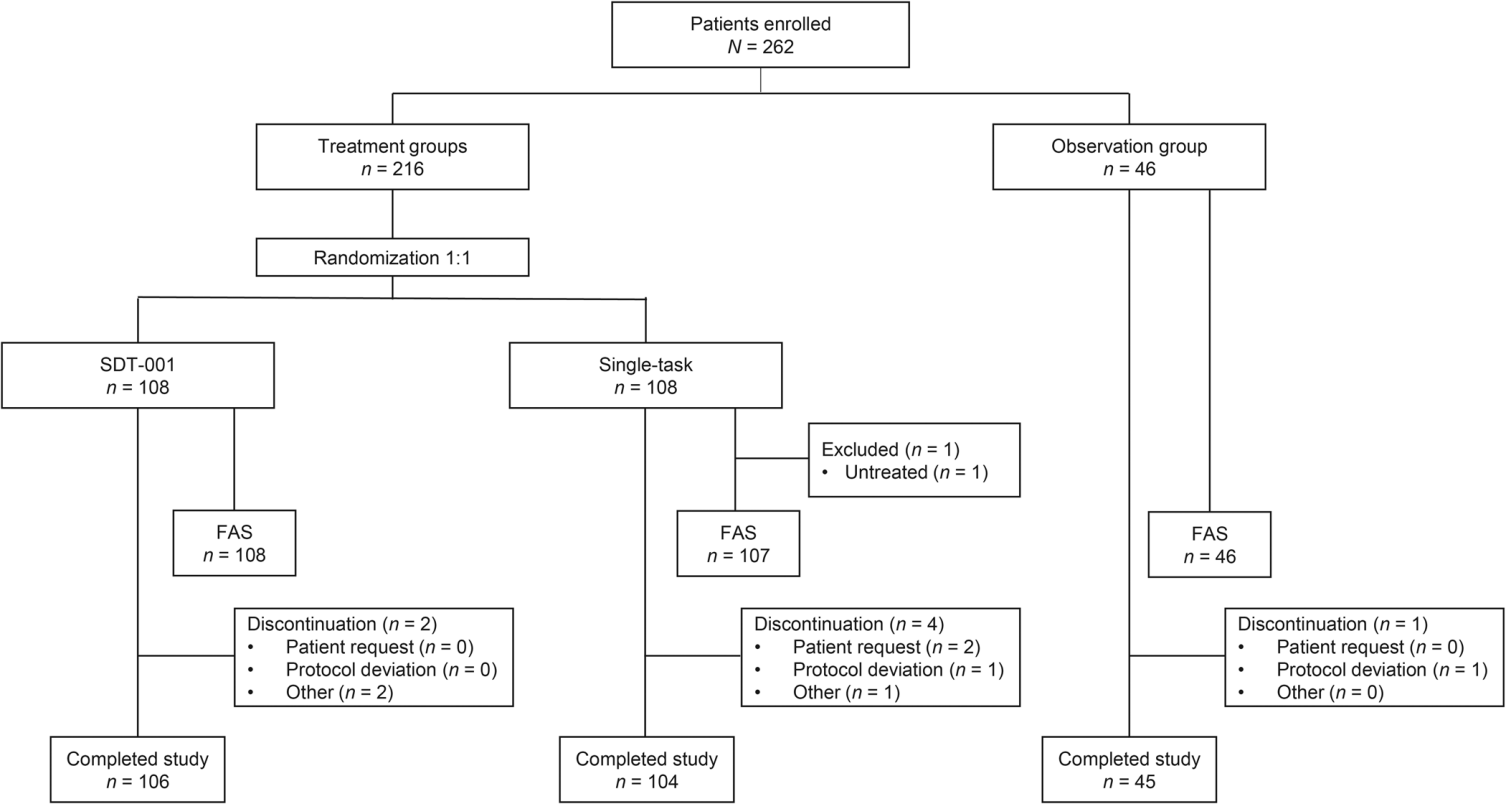
Patient disposition. FAS, full analysis set; SDT‐001, investigational digital therapeutic.

Overall, 262 participants were enrolled in the study between July 2020 and July 2021 and assigned to SDT‐001 (*n* = 108), single‐task (*n* = 108), or observation groups (*n* = 46) (Figure 2). Of these, 261 participants were included in the analysis (SDT‐001, *n* = 108; single‐task, *n* = 107; observation, *n* = 46). More participants discontinued in the single‐task group (3.7%, 4/108), followed by the observation (2.2%, 1/46) and SDT‐001 groups (1.9%, 2/108); reasons included participant's request, protocol deviation, and/or other.

### Patient demographic and baseline characteristics

The demographic and baseline clinical characteristics of the participants are summarized in Table 2. The ages (mean ± SD) of participants were 9.6 ± 2.3, 9.3 ± 2.1, and 9.0 ± 2.1 years, with the majority being male (73.1%, 77.6%, and 78.3%) in the SDT‐001, single‐task, and observation groups, respectively. Participants in the SDT‐001 group were predominantly inattentive (63.0%) and combined (37.0%); in the single‐task group, predominantly inattentive (52.3%), combined (46.7%), and hyperactive‐impulsive (0.9%); and in the observation group, predominantly inattentive (41.3%) and combined (58.7%) ADHD subtypes.

**Table 2:** Demographics and baseline characteristics of participants.

Characteristics	SDT‐001	Single‐task	Observation
	*n* = 108	*n* = 107	*n* = 46
Sex			
Male	79 (73.1)	83 (77.6)	36 (78.3)
Female	29 (26.9)	24 (22.4)	10 (21.7)
Age (years)			
Mean ± SD	9.6 ± 2.3	9.3 ± 2.1	9.0 ± 2.1
Median (min, max)	10.0 (6, 17)	9.0 (6, 16)	9.0 (6, 14)
≤12 years	98 (90.7)	98 (91.6)	43 (93.5)
≥13 years	10 (9.3)	9 (8.4)	3 (6.5)
Ethnicity			
Not Hispanic or Latino	108 (100.0)	107 (100.0)	46 (100.0)
Race			
Asian	107 (99.1)	107 (100.0)	46 (100.0)
Other	1 (0.9)	0	0
ADHD type			
Combined presentation	40 (37.0)	50 (46.7)	27 (58.7)
Predominantly inattentive presentation	68 (63.0)	56 (52.3)	19 (41.3)
Predominantly hyperactive‐impulsive presentation	0	1 (0.9)	0
ADHD‐RS‐IV inattention subscale score (physician's assessment)			
Mean ± SD	20.2 ± 3.1	20.2 ± 3.1	19.1 ± 3.4
Median (min, max)	20.0 (15, 27)	20.0 (15, 27)	18.5 (15, 26)
<19	36 (33.3)	38 (35.5)	23 (50.0)
≥19	72 (66.7)	69 (64.5)	23 (50.0)
TOVA® ACS			
Mean ± SD	−0.90 ± 3.71	−1.09 ± 3.37	−0.96 ± 3.62
Median (min, max)	−0.68 (−14.4, 6.0)	−1.23 (−12.6, 6.4)	−0.51 (−9.1, 5.1)
**<**0	59 (54.6)	65 (60.7)	29 (63.0)
≥0	46 (42.6)	42 (39.3)	17 (37.0)
Medication with indications for ADHD			
Yes	29 (26.9)	29 (27.1)	0
No	79 (73.1)	78 (72.9)	46 (100.0)
Psychosocial treatment including environmental coordination	108 (100.0)	107 (100.0)	46 (100.0)

*Note*: Data are presented as *n* (%) or mean ± SD unless specified.

Abbreviations: ACS, attention comparison score; ADHD, attention‐deficit/hyperactivity disorder; ADHD‐RS‐IV, ADHD rating scale‐IV; SD, standard deviation; SDT‐001, investigational digital therapeutic; TOVA, test of variables of attention.

At baseline, ADHD‐RS‐I (physician's assessment; mean ± SD) of participants was 20.2 ± 3.1 in both SDT‐001 and single‐task groups, and 19.1 ± 3.4 in the observation group. Most participants in SDT‐001 (66.7%) and single‐task groups (64.5%), and 50.0% in the observation group had ADHD‐RS‐I ≥19. The baseline TOVA® ACS (mean ± SD) of participants were −0.90 ± 3.71, −1.09 ± 3.37 and −0.96 ± 3.62 in SDT‐001, single‐task, and observation groups, respectively. In the SDT‐001 and single‐task groups, 26.9% and 27.1% of participants reported prior pharmacotherapy, respectively.

### Device exposure and compliance rate

The study device exposure period, defined as the total number of days the study device was used, (mean ± SD) was 37.8 ± 5.9 days for SDT‐001 and 38.0 ± 5.0 days for single‐task. The treatment compliance rate, referring to the proportion of daily use of the study device, was 87.9% for SDT‐001 and 89.4% for single‐task. The study device usage, defined as number of days used of the study device per week (mean ± SD) was 6.15 ± 0.91 days for SDT‐001 and 6.26 ± 0.69 days for single‐task (Table S3). In the blindness security assessment, 67.1% of participants and 58.5% of parents in the SDT‐001 group, and 62.5% and 51.5%, respectively, of those in the single‐task group responded to have used SDT‐001 (Table S4).

### Efficacy

**Figure 3:**
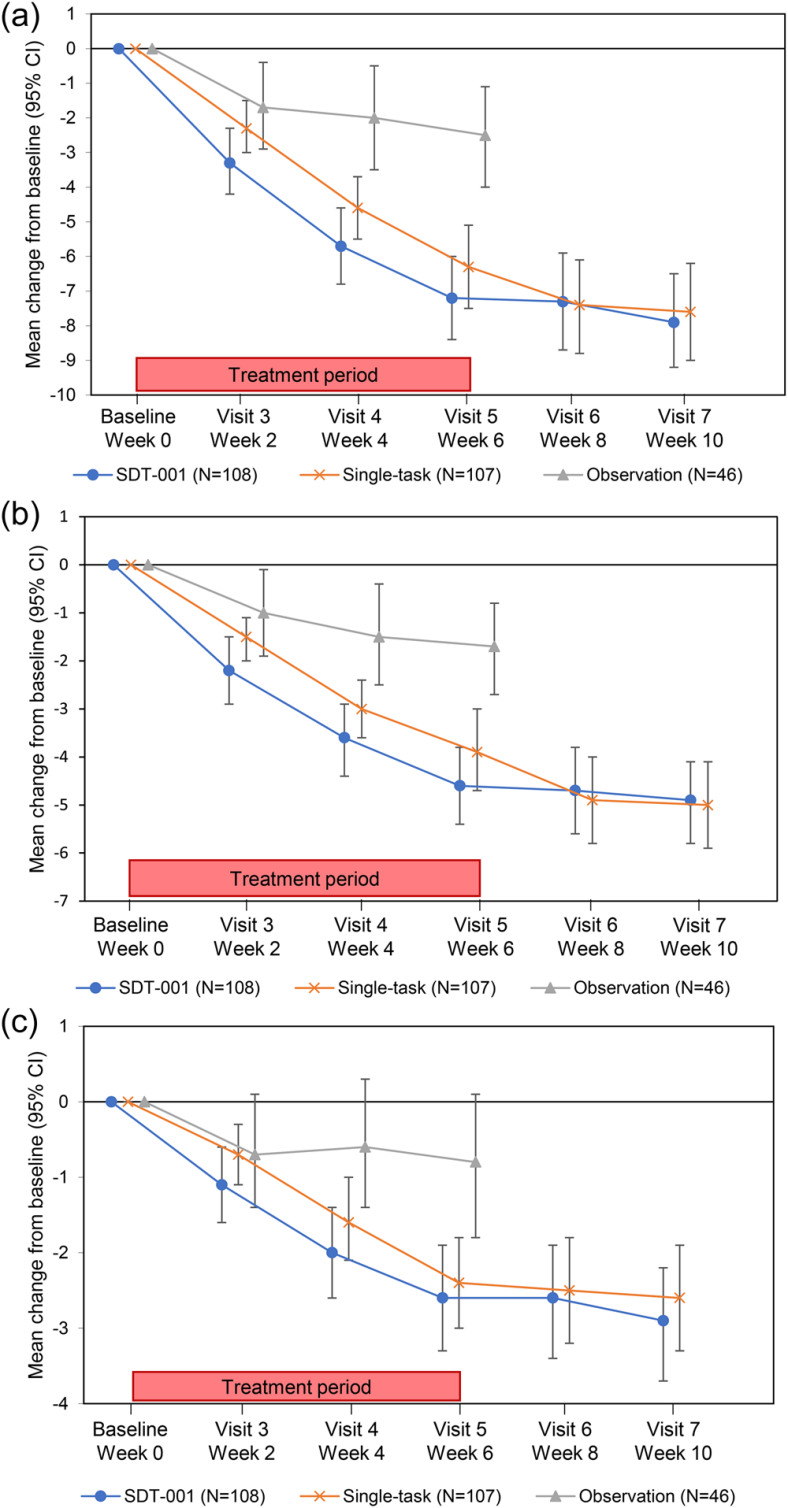
Change from baseline in ADHD‐RS‐IV (physician's) total score (a), inattention subscale score (b), and hyperactive‐impulsive score (c) during the treatment period and subsequent follow‐up period. The change from baseline to week 6 in ADHD‐RS‐IV score showed improvement with SDT‐001 use and remained stable during the 4‐week follow‐up period. ADHD‐RS‐IV, attention‐deficit/hyperactivity disorder rating scale‐IV; CI, confidence interval; SDT‐001, investigational digital therapeutic.

In the SDT‐001 group, ADHD‐RS‐IV (ADHD‐RS‐T, ADHD‐RS‐I, ADHD‐RS‐H; physician's assessment) decreased gradually from the beginning of usage (baseline) to week 6. Decreased scores were maintained for the next 4‐week follow‐up period (Figure 3a–c). Furthermore, the change from baseline to week 6 in ADHD‐RS‐IV scores (ADHD‐RS‐T, ADHD‐RS‐I, and ADHD‐RS‐H) was greater in the SDT‐001 group than in the single‐task group. However, the improvement in the SDT‐001 group was not statistically significant over the single‐task group at week 6 (SDT‐001 vs. single‐task: ADHD‐RS‐T: −7.5 [95% CI −9.0, −6.1] vs. −6.5 [−7.9, −5.0], P = 0.2112; ADHD‐RS‐I: −4.4 [−5.4, −3.4] vs. −3.6 [−4.6, −2.6], P = 0.1750; and ADHD‐RS‐H: −3.2 [−4.0, −2.5] vs. −2.9 [−3.6, −2.2], P = 0.4199). The changes from baseline to week 6 in TOVA® ACS, Conners 3™ (parents), IRS, CGI‐I, and PGA ratings with SDT‐001 and single‐task are summarized in Table S5.

In a post hoc analysis for participants without a history of pharmacotherapy, SDT‐001 or single‐task groups compared to the observation group showed a significant improvement in ADHD‐RS‐IV (physician's assessment) scores: ADHD‐RS‐T (SDT‐001 vs observation: −4.4 [−6.3, −2.6], P < 0.0001; single‐task vs. observation: −3.1 [−5.0, −1.2], P = 0.0013), ADHD‐RS‐I (SDT‐001 vs. observation: −2.5 [−3.7, −1.4], P < 0.0001; single‐task vs. observation: −1.7 [−2.9, −0.5], P = 0.0051), and ADHD‐RS‐H (SDT‐001 vs observation: −1.9 [−2.9, −0.8], P = 0.0005; single‐task vs. observation: −1.5 [−2.5, −0.4], P = 0.0077) (Table 3). In addition, IRS, Conners 3™ ADHD inattention (parents), Conners 3™ ADHD hyperactivity/impulsivity (parents), and CGI‐I ratings significantly improved in SDT‐001or single‐task groups as compared to the observation group, as summarized in Table S6.

**Table 3:** Comparison of changes in ADHD‐RS‐IV scores from baseline to week 6 (visit 5) in the efficacy outcomes (post hoc analysis).

Efficacy outcomes	SDT‐001 versus observation				Single‐task versus observation			
	Change from baseline to week 6		Average treatment effect/estimated difference between groups		Change from baseline to week 6		Average treatment effect/estimated difference between groups	
	SDT‐001 group (*n* = 78)	Observation group (*n* = 45)	Estimate [95% CI]	*p* value	Single‐task group (*n* = 76)	Observation group (*n* = 45)	Estimate [95% CI]	*p* value
ADHD‐RS‐IV total score (physician's)								
Estimate [95% CI]	−7.1 [−8.5, −5.8]	−2.7 [−3.9, −1.5]	−4.4 [−6.3, −2.6]	<0.0001	−5.7 [−7.2, −4.3]	−2.6 [−3.8, −1.3]	−3.1 [−5.0, −1.2]	0.0013
ADHD‐RS‐IV inattention score (physician's)								
Estimate [95% CI]	−4.4 −5.2, −3.6]	−1.9 [−2.8, −1.0]	−2.5 [−3.7, −1.4]	<0.0001	−3.5 [−4.4, −2.6]	−1.8 [−2.6, −0.9]	−1.7 [−2.9, −0.5]	0.0051
ADHD‐RS‐IV hyperactivity/impulsivity score (physician's)								
Estimate [95% CI]	−2.7 [−3.5, −1.9]	−0.8 [−1.6, −0.1]	−1.9 [−2.9, −0.8]	0.0005	−2.2 [−3.0, −1.5]	−0.8 [−1.6,0.0]	−1.5 [−2.5, −0.4]	0.0077

*Note*: Inverse probability weighting analysis using propensity score. Propensity score adjustment factors: age, sex, ADHD type, and baseline value of ADHD‐RS‐IV inattention score (physician's). In a post hoc analysis for the change in ADHD‐RS‐IV total and hyperactivity/impulsivity scores, the baseline value of the endpoint was included as a covariate in the model for the response variable. *p* values denote statistical significance at the P < 0.05 level.

Abbreviations: ADHD‐RS‐IV, attention‐deficit/hyperactivity disorder rating scale IV; CI, confidence interval; SDT‐001, investigational digital therapeutic.

### Safety

AEs and adverse device effects are summarized in Table 4. No deaths were reported during the study. One nonfatal serious adverse event (SAE) occurred in one participant in the single‐task group. No nonfatal serious adverse device effects occurred. More than one treatment‐emergent AE was reported in 35.2% of participants in the SDT‐001 group, 32.7% in the single‐task group, and 19.6% in the observation group. No AEs led to discontinuation of the study device.

**Table 4:** Adverse events and adverse device events classified by system organ class, preferred term in each category of severity.

Preferred term	SDT‐001	Single‐task	Observation
	*n* = 108	*n* = 107	*n* = 46
	*n* (%)	*n* (%)	*n* (%)
Patient with ≥1 TEAE	38 (35.2)	35 (32.7)	9 (19.6)
AEs ≥3/either of SDT‐001 or single‐task			
Nasopharyngitis	8 (7.4)	8 (7.5)	0 (0.0)
Headache	3 (2.8)	2 (1.9)	0 (0.0)
Somnolence	1 (0.9)	3 (2.8)	0 (0.0)
Pyrexia	3 (2.8)	1 (0.9)	0 (0.0)
Patient with ≥1 adverse device effect	4 (3.7)	4 (3.7)	–
Irritability	1 (0.9)	1 (0.9)	–
Headache	1 (0.9)	1 (0.9)	–
Somnolence	0 (0.0)	2 (1.9)	–
Eye asthenopia	0 (0.0)	1 (0.9)	–
Tinnitus	1 (0.9)	0 (0.0)	–
Nausea	1 (0.9)	0 (0.0)	–

*Note*: Multiple occurrences of an event for one participant are counted only once.

Includes only TEAEs. Medical Dictionary for Regulatory Activities version 23.0 is used for coding.

Abbreviations: AE, adverse event; SDT‐001, investigational digital therapeutic; TEAE, treatment‐emergent adverse event.

Adverse device effects occurred in 3.7% of participants in both SDT‐001 and single‐task groups (Table 4). Irritability (*n* = 1), headache (*n* = 1), tinnitus (*n* = 1), and nausea (*n* = 1) were the adverse device effects reported in the SDT‐001 group, with irritability (*n* = 1), headache (*n* = 1), somnolence (*n* = 2), and eye asthenopia (*n* = 1) reported in the single‐task group. One nonfatal SAE (humerus fracture) occurred in one participant in the single‐task group, which was not related to the study device. No nonfatal SAE occurred in the SDT‐001 and observation groups. Moderate AEs, injury, tooth fracture, and humerus fracture occurred in 1.9% of participants of the single‐task group (2/107), while avulsion fracture and defiant challenge disorder occurred in 1.9% of participants of the SDT‐001 group (2/108); these were not related to the study device.

For the questionnaire about gaming addiction, in the SDT‐001 and single‐task groups, 15.9% (17/107) and 15.2% (16/105) of participants, respectively, responded “I want to use the study device for a longer time in a day” at visit 5/week 6. In the SDT‐001 and single‐task groups, respectively, 35.5% (38/107) and 29.5% (31/105) of participants at visit 5/week 6 and 32.0% (33/103) and 27.7% (28/101) of participants at visit 7/follow‐up responded “I want to use the study device again.”

## DISCUSSION

This exploratory study assessed the efficacy and safety of SDT‐001, a DTx, in Japanese children and adolescents with ADHD. The active intervention, SDT‐001, showed improvement in ADHD‐RS‐T, ADHD‐RS‐H, and ADHD‐RS‐I scores (physician's assessment) compared to single‐task over 6 weeks. In the SDT‐001 group, reduction in scores were maintained during 4 weeks of follow‐up. A similar trend of improvement in Conners 3™ for parents, IRS, and CGI‐I ratings (except TOVA® ACS) was observed with the SDT‐001 group. However, the improvement was not significantly different between SDT‐001 and single‐task groups (P ≥ 0.05). In a post hoc analysis in participants without a history of pharmacotherapy, SDT‐001 or single‐task groups showed a significant improvement in ADHD‐RS‐IV scores (ADHD‐RS‐T, ADHD‐RS‐H, and ADHD‐RS‐I; physician's assessment), Conners 3™ for parents, IRS, and CGI‐I ratings compared to the observation group (with psychosocial therapies and environmental adjustment) (all P < 0.05). In this study, most children and adolescents with ADHD were able to perform SDT‐001 for 25 min daily for 6 weeks, which suggests favorable treatment compliance in real‐world clinical settings.

The development of DTx has gained increasing attention, while posing a major challenge in establishing optimal control devices for clinical evaluations.^18^ Therefore, the selection of suitable controls is crucial to ensure the quality of evidence. Given the difficulties in achieving both indistinguishability and functional dissimilarity from the test device, establishing an appropriate control device for a double‐blind study is challenging. In this study, to compare the effect of SDT‐001 we developed a sham candidate device that has activity elements similar to the dual‐task SDT‐001 but performs a single‐task with the closest possible resemblance to SDT‐001. This study also evaluated the effect of SDT‐001 with an observational follow‐up group as control, which acknowledged the recommendations of involvement of multiple control groups in comparison to the test intervention.^18^ Furthermore, blindness was ensured as most participants in the SDT‐001 and single‐task groups believed that the study device assigned to them was SDT‐001 (Table S4).

This study suggested an improvement in ADHD symptoms for subjects using a dual‐task DTx, SDT‐001. Previous evidence of neuroimaging techniques highlighted neural substrates involvement in cognitive‐motor interactions.^25^, ^26^ Functional magnetic resonance imaging revealed the activation of a right‐sided network during dual‐task training.^25^ Task‐switching training led to improvements in inhibitory control and verbal WM in children with ADHD and stable methylphenidate medication.^27^ Activation of the primary motor cortex and supplementary motor areas, areas responsible for planning, control, and execution of movements, has been observed during dual‐task training.^26^ Moreover, both focused and divided attention tasks engage a widespread, predominantly right‐sided network involving prefrontal and parietal structures, with increased activity and recruitment of left‐sided homologues under higher cognitive demands.^25^ Similarly, the SDT‐001 being a dual‐task device likely involves the activation of neuronal substrates engaged in cognitive‐motor interactions, with differences in activation patterns arising from varying demands of executive control as the task difficulty increases.^25^, ^26^ Moreover, SDT‐001 may modulate neural activity within this network to enhance cognitive‐motor performance. However, future research is needed to elucidate the specific mechanisms underlying this effect.

Post hoc analysis, in participants without a history of pharmacotherapy, of this study was limited to compare the SDT‐001 or single‐task groups with a non‐randomized observational group that received existing psychosocial therapies and environmental adjustments; however, it showed an improvement with SDT‐001 or single‐task compared to the observation group. The differences between the SDT‐001 and observation groups were significant not only in inattention but also in hyperactivity and impulsivity scores, which suggests the benefits of SDT‐001 over psychosocial and environment adjustment therapy in the treatment of ADHD symptoms.

Furthermore, clinical studies have demonstrated the superiority of pharmacotherapy versus placebo for improvement of ADHD symptoms; however, symptoms recurred rapidly on discontinuation of the drugs.^28^, ^29^, ^30^, ^31^, ^32^ In contrast, in this study, reductions in scores were sustained over 4 weeks after discontinuation of SDT‐001 (Figure 3a–c). Conventional ADHD medications primarily enhance neurotransmission of catecholamines (such as dopamine and norepinephrine),^33^ while the mechanism of action of SDT‐001 is different, possibly involving an activation pattern of neural substrates with cognitive‐motor interactions.^25^, ^26^, ^27^ Maintained reductions in scores after treatment discontinuation suggest a beneficial aspect from the treatment compliance perspective. In line with this study, a similar sustained effect has been observed with other digital interventions, such as AKL‐T01^16^ and Brain–Computer–Interface^34^ in children with ADHD, and NeuroRacer in the elderly.^13^ Earlier studies indicated that engaging in single‐task or dual‐task activates the prefrontal cortex,^13^, ^35^ which is believed to be involved in ADHD symptoms. However, investigations are needed to understand the mechanisms behind the sustained reductions in scores beyond 4 weeks. Specific mechanisms common to the SDT‐001 and single‐task groups may have resulted in improvements in both groups.

It is also essential to acknowledge the array of common and rare AEs associated with ADHD pharmacotherapy.^10^ In a systematic review, AEs such as nausea, vomiting, loss of appetite, insomnia, depressive symptoms, irritability, and social withdrawal were reported in children and adolescents treated with pharmacotherapy for ADHD.^36^ Conversely, in this study, SDT‐001 was well tolerated without any serious AEs in children and adolescents with ADHD. All device‐related AEs caused by SDT‐001 were mild, with no SAEs reported, which revealed the absence of safety concerns. Moreover, no adverse incidence of dependence/addiction of SDT‐001 was identified in this study.

In ADHD, psychosocial treatment may be perceived as safer therapy; however, the effects of psychosocial therapy showed variability between therapists/clinicians.^8^, ^9^ The use of a DTx such as SDT‐001 could aid in reducing the clinician‐induced instability in treatment responses and outcomes.

The results should be considered with some limitations of the study. The ADHD‐RS scores were slightly improved with SDT‐001 > single‐task > observation group, and the scores were greater with SDT‐001. Although the single‐task group could not be distinguished from SDT‐001 groups and ascertained double‐blind conditions, we speculate that it failed to play its role as a true sham control as it had some beneficial effect on ADHD‐RS scores. Thus, the findings of this study should be interpreted considering that the single‐task group is not likely to be a true sham‐controlled group. Indeed, similar to this study, several clinical studies and a meta‐analysis have demonstrated the effectiveness of DTx in a single‐task and dual‐task format, with a lack of difference between the single‐task and dual‐task groups.^11^, ^13^, ^15^, ^16^, ^17^, ^37^, ^38^, ^39^, ^40^

## CONCLUSION

The study showed improvement in inattention and hyperactivity/impulsivity with SDT‐001 compared to the single‐task group from baseline in children and adolescents with ADHD in Japan. Furthermore, the reductions in scores were sustained after discontinuation of SDT‐001 over 4 weeks. The SDT‐001 group showed a trend toward better improvement in ADHD‐RS‐IV scores (from baseline to week 6) than the single‐task group; the difference in improvement was not statistically significant (P > 0.05) between the SDT‐001 and single‐task groups. In a post hoc analysis in participants without a history of pharmacotherapy, SDT‐001 was found to be effective in improving ADHD symptoms compared to the observation group. Although this single‐task device was able to ensure blinding as a sham candidate, the results of the post hoc analysis comparing the single‐task group with the observation group showed a certain effect similar to the previous findings and the single‐task group; hence, a single assignment was not appropriate as the sham candidate. Further, clinical studies comparing SDT‐001 with appropriate controls are required to validate the efficacy of SDT‐001 in treating ADHD symptoms in the future. Overall, SDT‐001 could offer a novel treatment option, addressing the challenges associated with psychosocial treatment and pharmacotherapy in ADHD.

## AUTHOR CONTRIBUTIONS

Katsunaka Mikami, Tasuku Miyajima, Ryo Nishino, and Takashi Okada conceptualized, designed, and interpreted the results. Hiroki Fukuju conceptualized, designed, acquired, and analyzed the data. Naohiro Kawazoe and Toshimitsu Ochiai conceptualized, designed, and analyzed the data. All authors were involved in drafting and/or critically reviewing the manuscript and gave final approval of the version to be published. All authors agree to be accountable for all aspects of the work.

## CONFLICT OF INTEREST STATEMENT

Katsunaka Mikami has received financial support from Shionogi & Co., Ltd.; honoraria from Shionogi & Co., Ltd., Sumitomo Pharma Co., Ltd., and Takeda Pharmaceutical Co., Ltd.; travel and accommodation expenses from Otsuka Pharmaceutical Co., Ltd.; travel and accommodation expenses for the spouse from Pfizer; and a consulting fee from Shionogi & Co., Ltd., EA Pharma Co., Ltd., Sumitomo Pharma Co., Ltd., and Otsuka Pharmaceutical Co., Ltd. Tasuku Miyajima has received payment or honoraria for lectures, presentations, speakers bureaus, manuscript writing, or educational events from Nobelpharma Co., Ltd., Janssen Pharmaceutical K.K., and Shionogi & Co., Ltd.; and consulting fees from Shionogi & Co., Ltd. Takashi Okada has received payment or honoraria for lectures, presentations, speakers bureaus, manuscript writing, or educational events from Janssen Pharmaceutical K.K., Shionogi & Co., Ltd., Yoshitomi Yakuhin Co., Takeda Pharmaceutical Co., Ltd., and Nobelpharma Co., Ltd. Ryo Nishino declares no financial competing interests. Naohiro Kawazoe, Toshimitsu Ochiai, and Hiroki Fukuju are employees of Shionogi & Co., Ltd. and may hold stocks in the company. None of the authors declare any competing nonfinancial interests.

## ETHICS APPROVAL STATEMENT

This study was conducted in compliance with the consensus framework for ethical principles derived from international guidelines, including the Declaration of Helsinki and Council for International Organizations of Medical Sciences, International Ethical Guidelines, Ministerial Ordinance on Good Clinical Practice for Medical Devices, and applicable laws and regulations. The study was approved by the Institutional Review Boards/Independent Ethics Committees (Table S2).

## PATIENT CONSENT STATEMENT

Written informed consent was obtained from legally authorized representatives prior to the study commencement. Written informed assent was obtained from the participants themselves wherever possible.

## CLINICAL TRIAL REGISTRATION

The study was registered with the Japan Registry for Clinical Trials (jRCT) Clinical Trials Registry before patient enrollment (Study ID: jRCT1080225158).

## Supporting information

Supporting information.

## Data Availability

The datasets used and/or analyzed during the current study can be available from the corresponding author on reasonable request.
