# Supplementary material for: Exploratory study of dual‐task digital device in children and adolescents with attention‐deficit/hyperactivity disorder
Source: PCN Rep. 2025 Apr 2;4(2):e70089. doi: 10.1002/pcn5.70089 (PMC11964945; doi:10.1002/pcn5.70089)
Supplement: Supplementary file 1 — Supporting information. [file PCN5-4-e70089-s001.docx]

**Supplemental Material**

**Supplementary Table S1** Study eligibility criteria for participation

| Inclusion criteria | Patients meeting all the following inclusion criteria were eligible for the study |
| --- | --- |
| Age | 1. Outpatients who were pupils or students aged 6 years or older and younger than 18 years at the time of informed consent |
| Study population and disease characteristics | 1. Patients whose primary diagnosis^†^ based on the diagnostic criteria of the DSM-5 was attention-deficit/hyperactivity disorder (ADHD)  - 314.01 (F90.2) Combined - 314.00 (F90.0) Predominantly inattentive - 314.01 (F90.1) Predominantly hyperactive-impulsive |
|  | 1. Patients confirmed to have received psychosocial treatment (including environmental adjustment) for ADHD and were considered not to have a sufficient effect |
|  | 1. Patients had not received pharmacotherapy for ADHD within 7 days before informed consent. The observation group enrolled patients who had never received pharmacotherapy for ADHD |
|  | 1. Patients whose Attention Deficit/Hyperactivity Disorder Rating Scale IV (ADHD-RS-IV) inattentive subscale scores (physician’s assessment) at both visit 1 and visit 2 were ≥15 points |
|  | 1. Patients whose teachers could be requested to cooperate, and for whom the results of the teacher’s assessment could be confirmed at visit 2 |
| Sex | 1. Male and female |
| Informed consent | 1. Written consent to voluntary study participation could be obtained from the patient’s legally authorized representative. For patients aged 13 years or older, written informed assent was also to be obtained from patients themselves. For patients aged 6 to <13 years, written informed assent was to be obtained from patients themselves wherever possible. |
| Exclusion criteria | Patients meeting any of the following criteria were ineligible for the study |
| Medical conditions | 1. Patients with psychiatric disease such as schizophrenia spectrum, depression, or bipolar disorder; however, patients with concurrent autism spectrum disorder or localized learning disorder might be included |
|  | 1. Patients with personality disorder or intellectual disability or patients with suspected intellectual disability with an intelligence quotient of <70 in an intelligence test (or a previous intelligence test within the last 1 year, if any, and if acceptable in the opinion of the investigator) |
|  | 1. Patients concurrently or previously with convulsion or severe tic disorder (including Tourette’s disorder); however, patients concurrently or previously with febrile convulsion were eligible |
| Prior/concomitant therapy | 1. Patients who had used any prohibited concomitant drugs/therapies during the period from the time of informed consent to visit 2, or patients who received any additional psychosocial treatments (including environmental adjustment) or change in the conditions of treatment |
| Prior/concurrent clinical study experience | 1. Patients who have received or used any other investigational drug/study device within 90 days prior to visit 1 2. Patients who have participated in this study or patients whose siblings were participating in this study or who have participated in this study |
| Diagnostic assessments | 1. Patients whose change rates in the ADHD-RS-IV inattentive subscale score (physician’s assessment) at visit 2 exceeded 30% compared to that at visit 1 |
|  | 1. Patients who were considered unable to undergo assessments (e.g., the Test of Variables of Attention) requiring operation of the application and study-specific activities for physical and other reasons, e.g., deafness, color blindness, fractured hand, or arm |
|  | 1. Patients with suspected gaming disorder (playing games greatly interfered with everyday life [e.g., school life, sleep]) |
|  | 1. Patients with suicidal tendency meeting any of the following:  - A patient previously with suicide attempt - A patient concurrently or previously with suicidal ideation - A patient who had answered “Yes” to Question 4 or Question 5 of suicidal ideation or any questions of suicidal behavior in the Columbia-Suicide Severity Rating Scale within the last 6 months |
|  | 1. Patients with suspected substance-related disorder within 180 days before visit 1 |
| Other exclusion criteria | 1. Other patients who were considered by the investigator (sub-investigator) to be inappropriate for participation in the study, such as those who had difficulty in completing the study or making assessments, those who might be disadvantaged by participation in the study, or those for whom it was difficult to ensure safety during the study period |
| Definitions |  |
| Prior therapy | Therapies which were provided before informed consent  Therapies included pharmacotherapies for ADHD used before informed consent (regardless of indication for ADHD), and psychosocial treatments for ADHD (including environmental adjustments) used within 6 months before informed consent |
| Concomitant therapy | Therapies (including prescription drugs, vaccines, over-the-counter drugs, and health foods) provided from the time of informed consent to visit 7 or discontinuation (treatment period or follow-up period) |
| Prohibited concomitant therapy | The use of the following drugs (including prescription drugs, OTC drugs, and health foods) and therapies was prohibited from the time of informed consent to visit 7 or discontinuation (treatment period or follow-up period); however, drugs other than the investigational drug that were intended for external use or eye drops for local action might be used concomitantly   - Antipsychotic drugs - Selective noradrenaline reuptake inhibitors (e.g., atomoxetine hydrochloride) - Selective α2A adrenoceptor agonist (e.g., guanfacine hydrochloride) - Stimulants (e.g., methylphenidate hydrochloride, lisdexamfetamine mesilate) and antitussive drugs and cold drugs that contain such components - Depressants (e.g., sedatives, barbiturates) - Antidepressants - Mood stabilizers - Antianxiety drugs - Benzodiazepines - Appetite suppressants - Monoamine oxidase inhibitors - Anticonvulsants - Sedative antihistamines (including drugs containing sedative antihistamines) - Hypnotics - Other investigational drug/investigational device - Nutraceuticals that may have effects on the central nervous system (e.g., St. John’s wort, ginkgo leaf, kava, ephedra, melatonin) |
| Restricted concomitant therapy | Psychosocial treatment (including environmental adjustment) for ADHD that had been continued from before the consent might be continued during the study period; however, it was not allowed to add psychosocial therapies (including environmental adjustments) or change the conditions after informed consent |

^†^The disease chiefly requiring outpatient medical care in patients with more than one diagnosis.

**Supplementary Table S2** List of Institutional Review Boards (IRBs)

| **Study Center Name** | **Name of IRB / IEC** |
| --- | --- |
| Hokkaido University Hospital | Hokkaido University Hospital Institutional Review Board |
| Ujiie Memorial Clinic for Children | Sugiura Clinic Institutional Review Board |
| Kon Pediatric Clinic | Sugiura Clinic Institutional Review Board |
| Igarashi Pediatrics Clinic | Sugiura Clinic Institutional Review Board |
| Nanko kokorono Clinic | Nanko kokorono Clinic Institutional Review Board |
| Hitachinaka General Hospital | Review Board of Human Rights and Ethics for Clinical Studies Institutional Review Board |
| Tsuchiura Kyodo General Hospital | Review Board of Human Rights and Ethics for Clinical Studies Institutional Review Board |
| Ibaraki Children’s Hospital | Sugiura Clinic Institutional Review Board |
| Ken Clinic | Mizuo Clinic Institutional Review Board |
| Fukaya Mental Clinic | Shoda Hospital Institutional Review Board |
| Hello Clinic | Sugiura Clinic Institutional Review Board |
| Kohnodai Hospital,National Center for Global Health and Medicine | Kohnodai Hospital,National Center for Global Health and Medicine Institutional Review Board |
| Sotobou Kodomo Clinic | Dr.Mano Medical Clinic Institutional Review Board |
| Tokyo Women's Medical University Hospital | Tokyo Women's Medical University Institution Review Board |
| National Center of Neurology and Psychiatry | National Center of Neurology and Psychiatry Institutional Review Board |
| Kyorin University Hospital | Kyorin University Hospital Institutional Review Board |
| Shimada Ryoiku Medical Center for Challenged Children | Japan Conference of Clinical Research Institutional Review Board |
| Minami-aoyama Antique Street Clinic | Suzuki Internal & Circulatory Medical Clinic Institutional Review Board |
| Tokyo Nishi Tokushukai Hospital | Tokushukai Group Institutional Review Board |
| Oyamadai Suku-suku Clinic | Mizuo Clinic Institutional Review Board |
| Hattatsu Shinryo Clinic | Japan Conference of Clinical Research Institutional Review Board |
| Ohwa Mental Clinic | Yoyogi Mental Clinic Institutional Review Board |
| Aiiku Clinic | Suzuki Internal & Circulatory Medical Clinic Institutional Review Board |
| Medical corporation Nobita Micri Kids Clinic | Mizuo Clinic Institutional Review Board |
| Kodomo Mental Clinic Shinotsuka | Mizuo Clinic Institutional Review Board |
| Nobisuko Children Clinic | Mizuo Clinic Institutional Review Board |
| Kishiro Mental Clinic | Mizuo Clinic Institutional Review Board |
| Sinsenkokoro-clinic | Dr.Mano Medical Clinic Institutional Review Board |
| Yokohama Onoecho Clinic | Yoyogi Mental Clinic Institutional Review Board |
| Tsuji Pediatrics Clinic | Fukui General Hospital Institutional Review Board |
| University of Fukui Hospital | University of Fukui Hospital Institutional Review Board |
| Hiratani Children’s Clinic | Fukui General Hospital Institutional Review Board |
| Shinshu University Hospital | Shinshu University Hospital Institutional Review Board |
| Azumino Internal Medicine Stress Care Clinic | Review Board of Human Rights and Ethics for Clinical Studies Institutional Review Board |
| Tenryu Hospital | Tenryu Hospital Institutional Review Board |
| HAMAMATSU City Child Psychiatric Clinic | Tenryu Hospital Institutional Review Board |
| Mikawa Hospital | Dr.Mano Medical Clinic Institutional Review Board |
| Child Clinic PaPa | Sugiura Clinic Institutional Review Board |
| Sugawara Clinic | Nishi Hospital Institutional Review Board |
| Osaka Asahi Children's Hospital | Sugiura Clinic Institutional Review Board |
| Nishimura Peditric Clinic | Nihonbashi Sakura Clinic　Institutional Review Board |
| Osaka Psychiatric Medical Center | Osaka Psychiatric Medical Center Institutional Review Board |
| Yasuhara Children’s Clinic | Nihonbashi Sakura Clinic　Institutional Review Board |
| Mikunigaoka Kokoro Hospital | Japan Conference of Clinical Research Institutional Review Board |
| Takahashi Psychiatric Clinic | Tokyo Allergy and Respiratory Disease Research Institute Clinical Trial Review Committee |
| Hibiki Mental Clinic | Kondo Memorial Medical Foundation Tomisaka Clinic Institutional Review Board |
| Nara Medical University Hospital | Nara Medical University Hospital Institutional Review Board |
| Kyo Mental Clinic | Dr.Mano Medical Clinic Institutional Review Board |
| Minami Wakayama Medical Center | Minami Wakayama Medical Center Institutional Review Board |
| Hiroshima-Nishi Medical Center | Hiroshima-Nishi Medical Center Institutional Review Board |
| Mukainada ekimae Mental Clinic | Dr.Mano Medical Clinic Institutional Review Board |
| Murakawa clinic | Sugiura Clinic Institutional Review Board |
| Matsuyama Municipal Hospital | Matsuyama Municipal Hospital Institutional Review Board |
| Ehime University Hospital | Ehime University Hospital Institutional Review Board |
| Fukuoka University Hospital | Fukuoka University Hospital Institutional Review Board |
| Kaku Mental Clinic | Sugiura Clinic Institutional Review Board |
| Parkside Kokoronohattatsu Clinic | Nihonbashi Sakura Clinic　Institutional Review Board |
| Mental Clinic Iris | Dr.Mano Medical Clinic Institutional Review Board |
| Saga Medical and Welfare Center for the challenged | Saga Memorial Hospital　Institutional Review Board |
| Sagaekiminami Clinic | Sugiura Clinic Institutional Review Board |
| Arata Clinic | Sugiura Clinic Institutional Review Board |
| Shimodoori　Psychosomatic medical clinic | Dr.Mano Medical Clinic Institutional Review Board |
| Miyakonojo Shinsei Hospital | Sugiura Clinic Institutional Review Board |
| Chikama Clinic | Sugiura Clinic Institutional Review Board |
| Kagoshima Prefecture Children's General Rehabilitation center | Dr.Mano Medical Clinic Institutional Review Board |

**Supplementary Table S3** Study device exposure and compliance

|  | SDT-001 | Single-task |
| --- | --- | --- |
|  | *n* = 108 | *n* = 107 |
| Total number of days use of the study device (days) | | |
| Mean±SD | 37.8±5.9 | 38.0±5.0 |
| Proportion of use of the study device (%)^†^ | | |
| Mean±SD | 87.9±13.0 | 89.4±9.8 |
| <50 | 2 (1.9) | 0 (0.0) |
| ≥50 to <80 | 21 (19.4) | 17 (15.9) |
| ≥80 | 85 (78.7) | 90 (84.1) |
| Number of days used of the study device per week (days/week) ^‡^ | | |
| Mean±SD | 6.15±0.91 | 6.26±0.69 |

Data are mean±SD or *n* (%) unless specified.

^†^((number of days that completed 5 sessions) / (the latest date among the last date reported for status of use, visit 5, or discontinuation - the earliest date among the first date reported or visit 2 + 1)) × 100

^‡^ ((Proportion of use of the study device) / 100) x 7

SD, standard deviation; SDT-001, investigational digital therapeutic.

**Supplementary Table S4** Questionnaire about security of blindness (parents and participants)

|  | SDT-001 | Single-task |
| --- | --- | --- |
|  | *n* = 108 | *n* = 107 |
| Parents who guessed the study device assigned was SDT-001 | | |
| Total, *n* | 106 | 103 |
| Yes | 62 (58.5) | 53 (51.5) |
| No | 44 (41.5) | 50 (48.5) |
| Participants who guessed the study device assigned was SDT-001 | | |
| Total, *n* | 79 | 80 |
| Yes | 53 (67.1) | 50 (62.5) |
| No | 26 (32.9) | 30 (37.5) |

Data are *n* or *n* (%) unless specified.

SDT-001, investigational digital therapeutic.

**Supplementary Table S5** Comparison of changes from baseline to week 6 (visit 5) in the efficacy outcomes

| Outcomes | SDT-001 | | Single-task | | SDT-001 *vs* Single-task |
| --- | --- | --- | --- | --- | --- |
| Changes from baseline to week 6 (visit 5) | *n* | Least-square mean [95% CI] | *n* | Least-square mean [95% CI] | Difference [95% CI];  *P* value |
| TOVA^®^ ACS | 104 | -1.29 [-2.13, -0.45] | 105 | -2.08 [-2.92, -1.25] | 0.80 [-0.15, 1.74]; 0.1003 |
| Conners 3™ ADHD inattention subscale score (parents) | 107 | -3.1 [-4.4, -1.9] | 105 | -2.6 [-3.8, -1.4] | -0.5 [-1.8, 0.7]; 0.3824 |
| Conners 3™ ADHD hyperactivity/impulsivity subscale score (parents) | 107 | -3.1 [-4.3, -1.9] | 105 | -3.0 [-4.2, -1.8] | 0.0 [-1.2, 1.2]; 0.9652 |
| IRS | 107 | -0.8 [-1.1, -0.6] | 105 | -0.7 [-0.9, -0.4] | -0.2 [-0.5, 0.1]; 0.2795 |
| Percentage improvement | | | | | |
| CGI-I | 24.3% (26/107)  [16.5%, 33.5%] | | 21.0% (22/105)  [13.6%, 30.0%] | | 3.5% [-7.7%, 14.7%]; 0.5404 |
| PGA | 25.2% (27/107)  [17.3%, 34.6%] | | 19.0% (20/105)  [12.0%, 27.9%] | | 6.4% [-4.7%, 17.4%]; 0.2668 |

Data are least-square mean [95% CI] or percentage improvement. For Conners 3™, analysis of covariance with baseline value, age (category), and presence of previous medication with indications for ADHD as covariates. For TOVA**^®^** ACS and IRS, the linear model is applied, including treatment group, visit, treatment group-by-visit as fixed effects, and baseline value, age (category), and presence of previous medication with indications for ADHD as covariates, and the variance-covariance between times matrix is unstructured. Then the degree of freedom is adjusted with the Kenward and Roger method. For CGI-I and PGA, the Cochran-Mantel-Haenszel method is stratified by age (category) and medication with indications for ADHD.

ACS, attention comparison score; ADHD, attention-deficit/hyperactivity disorder; CGI-I, clinical global impression–improvement; CI, confidence interval; IRS, impairment rating scale; PGA, physician’s global assessment; SDT-001, investigational digital therapeutic; TOVA, test of variables of attention.

**Supplementary Table S6** Comparison of changes from baseline to week 6 (visit 5) in the efficacy outcomes (post hoc analysis)

|  | SDT-001 *vs* observation | | | | Single-task *vs* observation | | | |
| --- | --- | --- | --- | --- | --- | --- | --- | --- |
| Efficacy outcomes | Change from baseline to week 6 | | Average treatment effect/ estimated difference between groups | | Change from baseline to week 6 | | Average treatment effect/ estimated difference between groups | |
|  | SDT-001 group | Observation group | Estimate [95% CI] | *P* value | Single-task group | Observation group | Estimate [95% CI] | *P* value |
| TOVA^®^ ACS | | | | | | | | |
| *n* | 76 | 45 |  |  | 76 | 45 |  |  |
| Estimate  [95% CI] | -0.60 [-1.39, 0.19] | -0.76 [-1.74, 0.22] | 0.16 [-1.08, 1.40] | 0.7975 | -1.69 [-2.54, -0.85] | -0.86 [-1.88, 0.17] | -0.84 [-2.16, 0.48] | 0.2131 |
| IRS | | | | | | | | |
| *n* | 78 | 45 |  |  | 76 | 45 |  |  |
| Estimate  [95% CI] | -0.8 [-1.1, -0.5] | -0.1 [-0.4, 0.2] | -0.7 [-1.1, -0.2] | 0.0016 | -0.6 [-0.9, -0.4] | -0.1 [-0.4, 0.2] | -0.6 [-1.0, -0.1] | 0.0082 |
| Conners 3™ ADHD inattention (parents) | | | | | | | | |
| *n* | 78 | 45 |  |  | 76 | 45 |  |  |
| Estimate  [95% CI] | -3.3 [-4.3, -2.3] | -0.6 [-1.8, 0.5] | -2.6 [-4.1, -1.2] | 0.0004 | -3.0 [-3.8, -2.1] | -0.4 [-1.6, 0.7] | -2.5 [-4.0, -1.1] | 0.0005 |
| Conners 3™ ADHD hyperactivity/impulsivity (parents) | | | | | | | | |
| *n* | 78 | 45 |  |  | 76 | 45 |  |  |
| Estimate  [95% CI] | -2.9 [-3.9, -1.9] | -0.7 [-1.7, 0.3] | -2.2  [-3.7, -0.8] | 0.0025 | -3.1 [-4.1, -2.1] | -0.6 [-1.7, 0.6] | -2.6 [-4.1, -1.1] | 0.0008 |
| CGI-I | | | | | | | | |
| *n* | 78 | 45 |  |  | 76 | 45 |  |  |
| Estimated improvement  [95% CI] | 22.3% [13.0%, 31.6%] | 6.7% [-0.3%, 13.7%] | 15.6% [3.8%, 27.4%] | 0.0095 | 21.2% [11.9%, 30.6%] | 6.3% [-0.3%, 13.0%] | 14.9% [3.5%, 26.3%] | 0.0103 |
| PGA | | | | | | | | |
| *n* | 78 | 45 |  |  | 76 | 45 |  |  |
| Estimated improvement  [95% CI] | 24.6% [15.1%, 34.1%] | 15.9% [4.8%, 27.0%] | 8.7% [-5.8%, 23.3%] | 0.2406 | 18.4% [9.6%, 27.2%] | 14.3% [4.3%, 24.4%] | 4.0% [-9.3%, 17.3%] | 0.5524 |

Inverse probability weighting analysis using propensity score. Propensity score adjustment factors: age, sex, ADHD type, and baseline value of ADHD-RS-IV (physician’s assessment) inattentive subscale score. The baseline value of each endpoint was included as a covariate in the model for the change, except for a post hoc analysis for CGI-I and PGA. Bold values denote statistical significance at the *P*<0.05 level.

ACS, attention comparison score; ADHD-RS-IV, attention-deficit/hyperactivity disorder rating scale IV; CGI-I, clinical global impression–improvement; CI, confidence interval; IRS, impairment rating scale; PGA, physician’s global assessment; SD, standard deviation; SDT-001, investigational digital therapeutic; TOVA, test of variables of attention.
